# Integrated Analysis of Transcriptome and Metabolome Reveals the Mechanism of Chlorine Dioxide Repressed Potato (*Solanum tuberosum* L.) Tuber Sprouting

**DOI:** 10.3389/fpls.2022.887179

**Published:** 2022-05-23

**Authors:** Xiaoyuan Zheng, Mei Li, Shilong Tian, Shouqiang Li, Jianxin Chen, Xuejiao Zhang, Xiaohua Wu, Xia Ge, Jiachun Tian, Yuwen Mu, Juan Song

**Affiliations:** ^1^Agricultural Product Storage and Processing Research Institute, Gansu Academy of Agricultural Sciences, Lanzhou, China; ^2^College of Food Science and Engineering, Gansu Agricultural University, Lanzhou, China; ^3^Gansu Innovation Center of Fruit and Vegetable Storage and Processing, Lanzhou, China

**Keywords:** potato tuber, chlorine dioxide, repression of sprout, transcriptome, metabolome

## Abstract

Sprouting is an irreversible deterioration of potato quality, which not only causes loss in their commercial value but also produces harmful toxins. As a popular disinfectant, ClO_2_ can inhibit the sprouting of potato tubers. Using transcriptomic and metabolomic approaches to understand the repressive mechanism of ClO_2_ in potato sprouting is yet to be reported. Sequencing the transcriptome and metabolome of potatoes treated with ClO_2_ in this study revealed a total of 3,119 differentially expressed genes, with 1,247 and 1,872 genes showing down- and upregulated expression, respectively. The majority of the downregulated genes were associated with plant hormone signal transduction, whereas upregulated differential genes were associated primarily with biological processes, such as phenylpropanoid biosynthesis and the mitogen-activated protein kinase (MAPK) signaling pathway. Metabonomic assays identified a total of 932 metabolites, with 33 and 52 metabolites being down- and upregulated, respectively. Downregulated metabolites were mostly alkaloids, amino acids, and their derivatives, whereas upregulated metabolites were composed mainly of flavonoids and coumarins. Integrated transcriptomic and metabolomic analyses showed that many different metabolites were regulated by several different genes, forming a complex regulatory network. These results provide new insights for understanding the mechanism of ClO_2_-mediated repression of potato sprouting.

## Introduction

Potato is one of the most important food crops in the world, with a high nutritional value. However, its postharvest storage remains a challenging step to maintaining quality and minimizing market losses (Huang et al., 2014). During the potato storage in the fresh food market, or storage of tubers for seed harvesting and industrial processing, sudden sprouting occurs (Teper-Bamnolker et al., 2010). Tuber sprouting is controlled by several factors, such as plant hormones, genetic factors, signaling molecules, genotype, temperature, and other storage conditions (Wang et al., 2020). Sprouting is also determined by metabolic activities that regulate phytohormones to exert growth control (Sonnewald, 2001).

Potato sprouting causes undesirable tuber weight loss, decay, and overall quality decline in fresh food and seed tubers, leading to significant market losses (Kleinkopf et al., 2003). In addition, sprouting increases the accumulation of solanine in tubers, this might cause mild to acute poisoning when the content exceeds 200 mg/kg (Liu et al., 2020). Thus, the application of sprout inhibitors is necessary during the storage of potato tubers to avoid postharvest losses. Chlorpropham is registered as a commercial product that has been shown to have the best sprout-repressive effect on potato tubers (Corsini et al., 1978; Campbell et al., 2010). However, residual chlorpropham in the treated seed tubers inhibited field sprouting; thus, developing an effective potato sprouting suppressant is an urgent research target (Conte et al., 1995).

Chlorine dioxide (ClO_2_) has broad and high-biocidal activity because of its strong oxidizing and sanitizing properties (Aday and Caner, 2011). ClO_2_ was cleared by the Food and Drug Administration (FDA (Food Drug Administration)., 1998) as a safe antibacterial agent for cleaning and treating fruits and vegetables. Chlorine significantly reduces sprouting and effectively controls dry rot, soft rot, and silver scurf of the tubers (Tweddell et al., 2003). For example, cleaning fruit surfaces with ClO_2_ reduced air-dried *Salmonella* in tomato and *Escherichia coli* O157:H7 strain in apples (Du et al., 2003; Pao et al., 2009). In addition, ClO_2_ treatment maintained quality, reduced rot rate, and extended the shelf life of kiwifruit (Niu et al., 2009), mulberry (Chen et al., 2011), and litchi (Wu et al., 2011), and reduced pericarp browning in longan (Atinut et al., 2019).

Currently, most studies are focused on the use of ClO_2_ in the control of pathogenic microbes, maintenance of fruit quality, and extending the shelf life of fruit. However, no studies on the repressive mechanism of ClO_2_ in potato sprouting using transcriptome and metabolome approaches have been reported. This study analyzed the transcriptomic and metabolic profiles of ClO_2_-treated tubers to uncover the molecular mechanism of ClO_2_-mediated repression of potato sprouting.

## Materials and Methods

### Materials

The tubers of the “Atlantic” cultivar were obtained from Kaikai Potato Seed Co., Ltd. (Gansu, China). After discarding the tubers with injuries or infections, potatoes were transferred to the laboratory and stored at 20 ± 3°C until use. A formulation with 12% ClO_2_ as the active ingredient was purchased from Tianjin Zhangda Technology Development Co., Ltd. (Tianjin, China).

### Treatment of Tubers and Sampling

After dormancy, tubers were cleaned and disinfected by immersion in 1% sodium hypochlorite for 3 min, rinsed with sterile water, and air-dried. Tubers were then soaked in 180 mg/L ClO_2_H_3_ for 15 min, and a control group (CKH3) was soaked in sterile water. After drying, all treated tubers were stored in the dark at 20 ± 2°C with a relative humidity of 75–85%. Experiments were carried out in three replicates per treatment, with each containing 30 tubers.

### Methods

#### Sampling

Sampling of the ClO_2_H_3_-treated tubers with no sprouts was performed after detecting sprouting in the CKH3 treatments. About 5 g of tuber tissue was cut 1 cm deep under the skin at the bud growth site (bud eye), with a 1 cm stainless steel punch. Samples were wrapped in tin foil paper, frozen in liquid nitrogen, and stored at −80°C until use.

#### RNA Sequencing (RNA-Seq)

Total RNA extraction (three duplicate samples) was performed following the method described by Woolfson et al. (2018). cDNA libraries were developed in three duplicates as described by Foucart et al. (2006). Libraries were sequenced using the Illumina HiSeq 2500 platform (Jisi Huiyuan Biotechnology Co., Ltd., Nanjing, China), with 2 × 150 paired-end reads. The high-quality reads were assembled into unigenes following the methods by Haas et al. (2013). To count the assemblies, TopHat 2 software was used to map RNA sequence data to the *Solanum tuberosum* reference genome (DM_1-3_516_R44_potato.v6.1), downloaded from the Potato Genomics Resource (http://spuddb.uga.edu/dm_v6_1_download.shtml).

#### Functional Annotation

The unigenes were queried against the Kyoto Encyclopedia of Genes and Genomes (KEGG), Non-redundant (Nr), Eukaryotic Orthologous Groups of protein (KOG), Uniprot, and Gene Ontology (GO) public databases, with a threshold e-value of <10^−5^ to obtain the functional annotations. The KEGG pathway analyses and GO functional classifications were visualized using the Web Gene Ontology Annotation Plot (WEGO) and KEGG automatic annotation servers, respectively.

#### Metabolite Extraction

The frozen samples were ground in a mixer mill for 1 min at 45 Hz. Approximately, 100 mg of samples were then mixed with 1.5 mL methanol–water (3:1). The samples were homogenized and sonicated for 15 min at 0°C, extracted with a shaker for 24 h at 4°C, followed by centrifugation with 13,800 × *g* at 4°C for 15 min. The supernatant was filtered through a membrane, the filtrate was diluted twice with methanol–water (3:1), and then transferred to glass vials; 20 μl of this solution was pooled as QC sample for ultra-high-performance liquid chromatography-mass spectrometry (UHPLC-MS) analyses (Sawada et al., 2009).

#### UHPLC-MS Analyses

Ultra-high-performance liquid chromatography-mass spectrometry separation was performed using an EXIONLC System, with mobile phases A and B as formic acid (0.1%) and acetonitrile, respectively. The column and the auto-sampler temperatures were set at 40°C. A Sciex QTrap 6500+ (Sciex Technologies) was applied. Ion source parameters were set as follows: temperature, 400°C; curtain gas, 35 psi; ion source gas 2, 60 psi; DP, ±100 V; ion source gas, 1:60 psi; and ion spray voltage, +5,500/−4,500 V (Zhang et al., 2015). The UHPLC-MS measurements were carried out in five replicates per sample.

#### Data Pre-Processing, Annotation, and Screening of Differentially Accumulated Metabolites

The MRM data were acquired with the SCIEX Analyst Work Station Software (Version 1.6.3), then the raw (.wiff) MS data files were converted to.txt format using the MSconverter. MS peak detection and annotation were performed in the R program (Kuhl et al., 2012). Differentially accumulated metabolites (DAMs) obtained were screened with pairwise comparison using the Variable Importance in the Projection (VIP) with the first principal component of the OPLS-DA model as >1 and *p* < 0.05, Student's *t*-test, as the threshold.

#### qRT-PCR Analyses

qRT-PCR analyses were performed following the methods of Woolfson et al. (2018), with the same RNA and cDNA samples used in the RNA-seq analysis. The accession numbers of selected genes based on the Potato Genomics Resource assembly database and their specific primers are presented in Supplementary Table 1. The primers for the housekeeping gene, *ef1*, were designed according to Nicot et al. (2005). The RT-qPCR reaction system was prepared as follows: Ultra SYBR mixture, 10 μl; RNase-free water, 8.4 μl; template cDNA, 0.8 μl; and each primer, 0.4 μl. RT-qPCR was performed with the Roche LightCycler 480 (Roche, Bazel, Switzerland) thermocycler, with the program set as follows: initial 95°C for 10 min, followed by 40 reaction cycles of 95°C for 30 s, 72°C for 30 s, one final cycle of 72°C for 10 min, and one cycle of 40°C for 30 s. The relative gene expression was calculated using the 2^−ΔΔCt^ method (Livak and Schmittgen, 2001).

## Results

### Quality Assessments of Sequencing Data and Differential Gene Expression Analyses

A total of 161.77 GB quality clean sequencing reads were obtained, with an average of 5.98 GB clean data per sample, and percentages Q20 and Q30 of bases above 97 and 93%, respectively, were observed. The guanine–cytosine (GC) content of each sample was above 42% after filtration (Supplementary Table 2). All clean reads were compared to the potato reference genome (DM_1-3_516_R44_potato.v6.1), and 89.25–89.59% or 89.25–90.10% of sequences from control or ClO_2_ treatments were mapped to the reference genome, which demonstrated the high reliability of our data (Supplementary Table 3).

**Figure 1:**
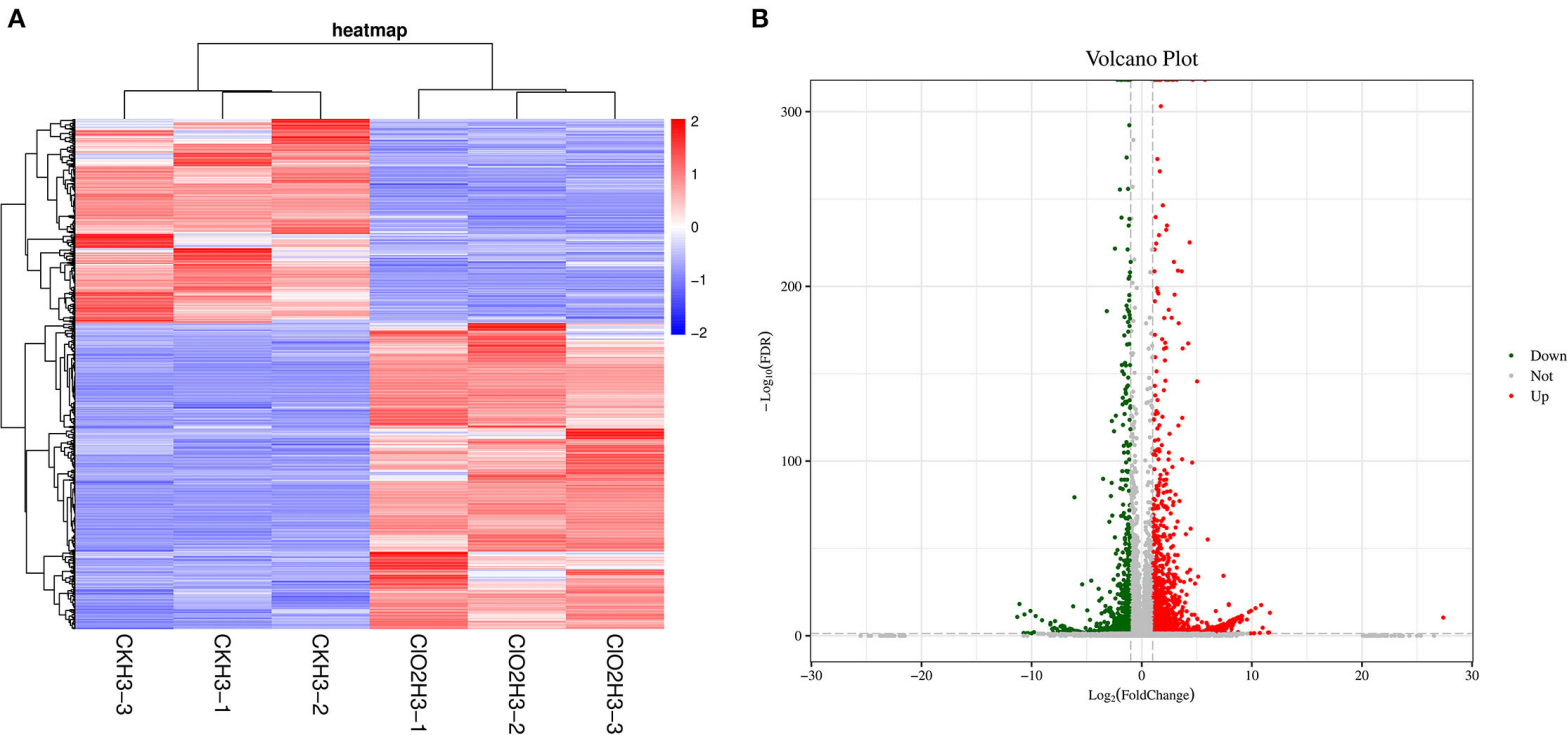
The cluster heat map of the differentially expressed genes **(A)** and volcanic map **(B)** in control and ClO_2_ treated tubers. Three biological repeats were shown in the figure. CKH3 represents the tubers of safflower treated with sterile water; ClO_2_H_3_ represents the flowers of safflower treated with ClO_2_.

To further investigate the gene expression patterns, differentially expressed genes (DEGs) were screened by pairwise comparison with Fold Change (FC) ≥2 and False Discovery Rate (FDR) <0.05 and Fragments per Kilobase of transcript per Million fragments mapped (FPKM) values were calculated for each sample to normalize the gene expression. Hierarchical clustering analysis was performed on the filtered differentially expressed genes, and the genes with the same or similar expression behaviors were clustered, and the results of the clustering of differentially expressed genes are shown in Figure 1A. A total of 3,119 DEGs were detected, with 1,247 and 1,872 as down- and upregulated genes, respectively, after ClO_2_ treatment (Figure 1B).

### GO Annotation and Enrichment

**Figure 2:**
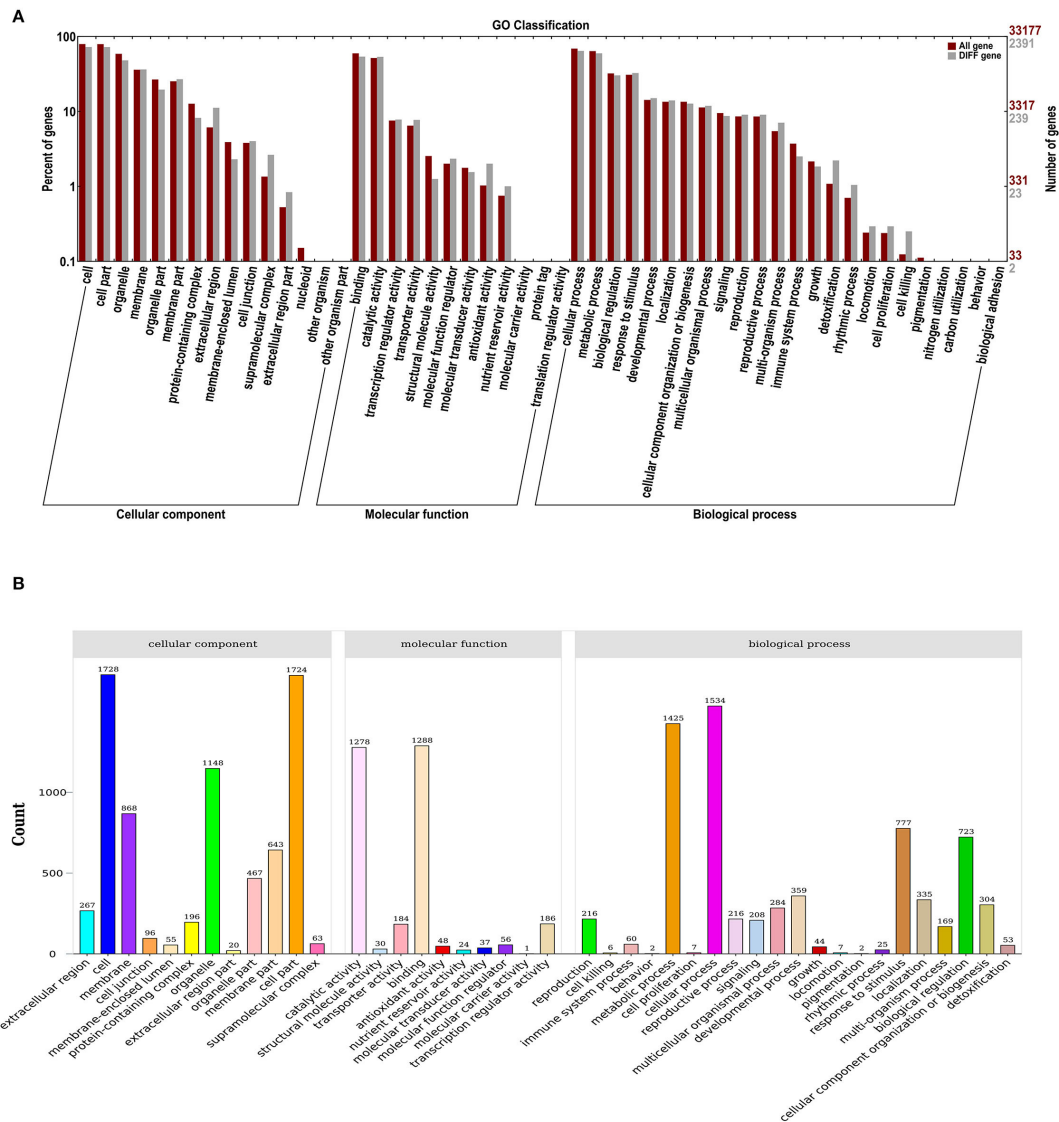
GO enrichment analysis **(A)** and statistical classification analysis **(B)** of differentially expressed genes.

GO classification was used to define the functions of DEGs from the comparisons between control and ClO_2_. As a result, 43 functional DEG categories were identified, including 12 “cellular components”, 10 “molecular functions”, and 21 “biological processes” (Figure 2A). Most DEGs were classified into the cell (1,728) and cell part (1,724) in the cellular components, whereas 1,288, 1,278, 1,534, and 1,425 DEGs were classified in binding, catalytic activity, molecular functions and cellular processes, and metabolic processes, respectively (Figure 2B).

### KEGG Annotation and Enrichment

**Figure 3:**
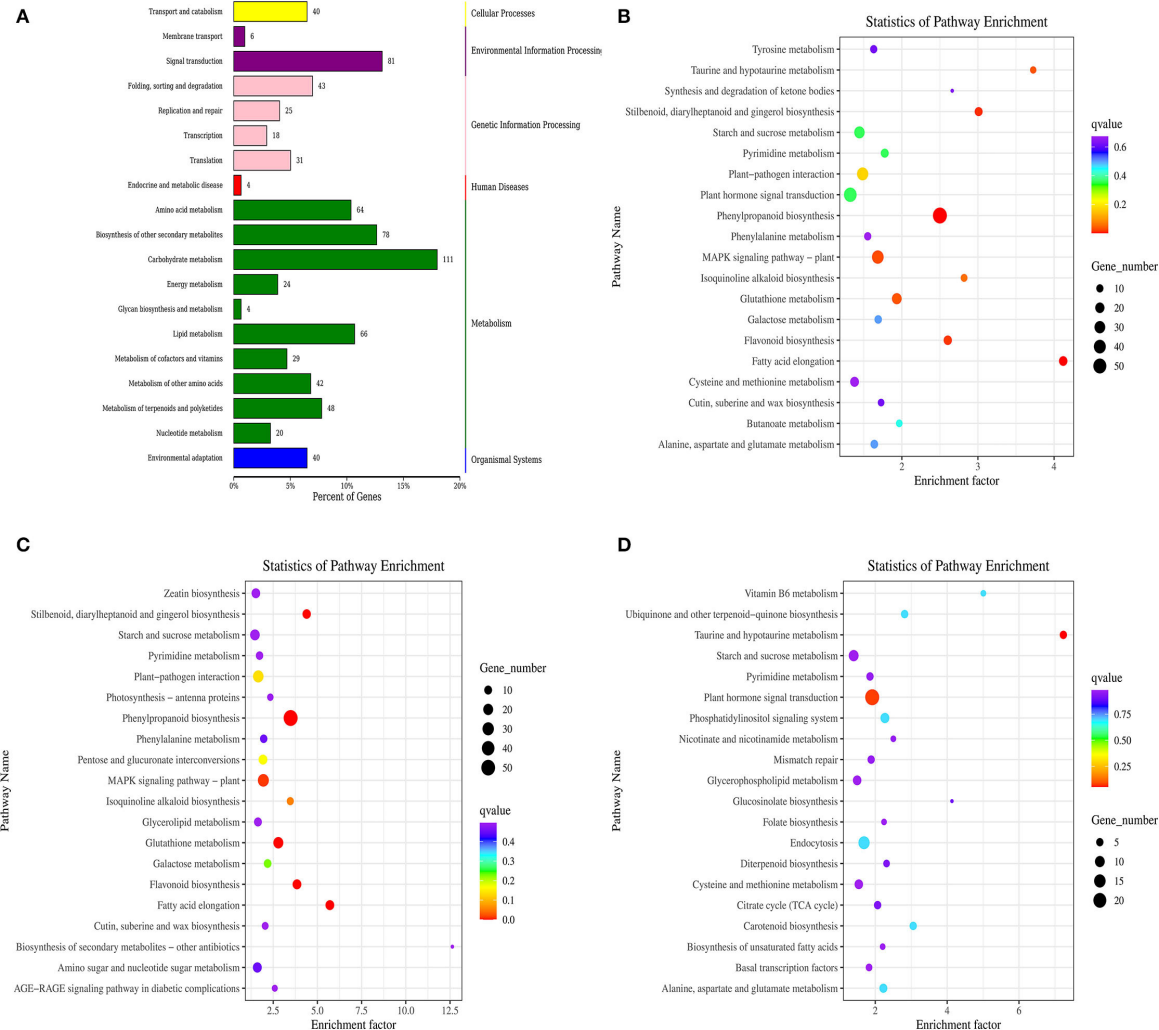
KEGG classification of differentially expressed genes **(A)** and KEGG pathway enrichment scatter of differentially expressed **(B)**, up-regulated gene **(C)**, and down-regulated gene **(D)**.

DEGs were screened in the KEGG ortholog database for functional enrichment analyses. As a result, the comparison between the control and ClO_2_-treated tubers assigned 19 pathways to different categories, including organismal systems, metabolism, environmental information processing, genetic information, cellular processes, and human diseases. In addition, carbohydrate metabolism, signal transduction, biosynthesis of other secondary metabolites, amino acid metabolism, and lipid metabolism were significantly enriched in DEGs (Figure 3A).

Notably, abundant DEGs were involved in phenylpropanoid biosynthesis, MAPK signaling pathway-plant, plant hormone signal transduction, plant–pathogen interaction, starch and sucrose metabolism, and glutathione metabolism(Figure 3B). Interestingly, only the plant hormone signal transduction pathway was enriched with downregulated genes, whereas the other two pathways were enriched with upregulated genes (Figures 3C,D).

### Analyses of DEG in Phenylpropanoid Biosynthesis and Plant Hormone Signal Transduction Pathways

**Figure 4:**
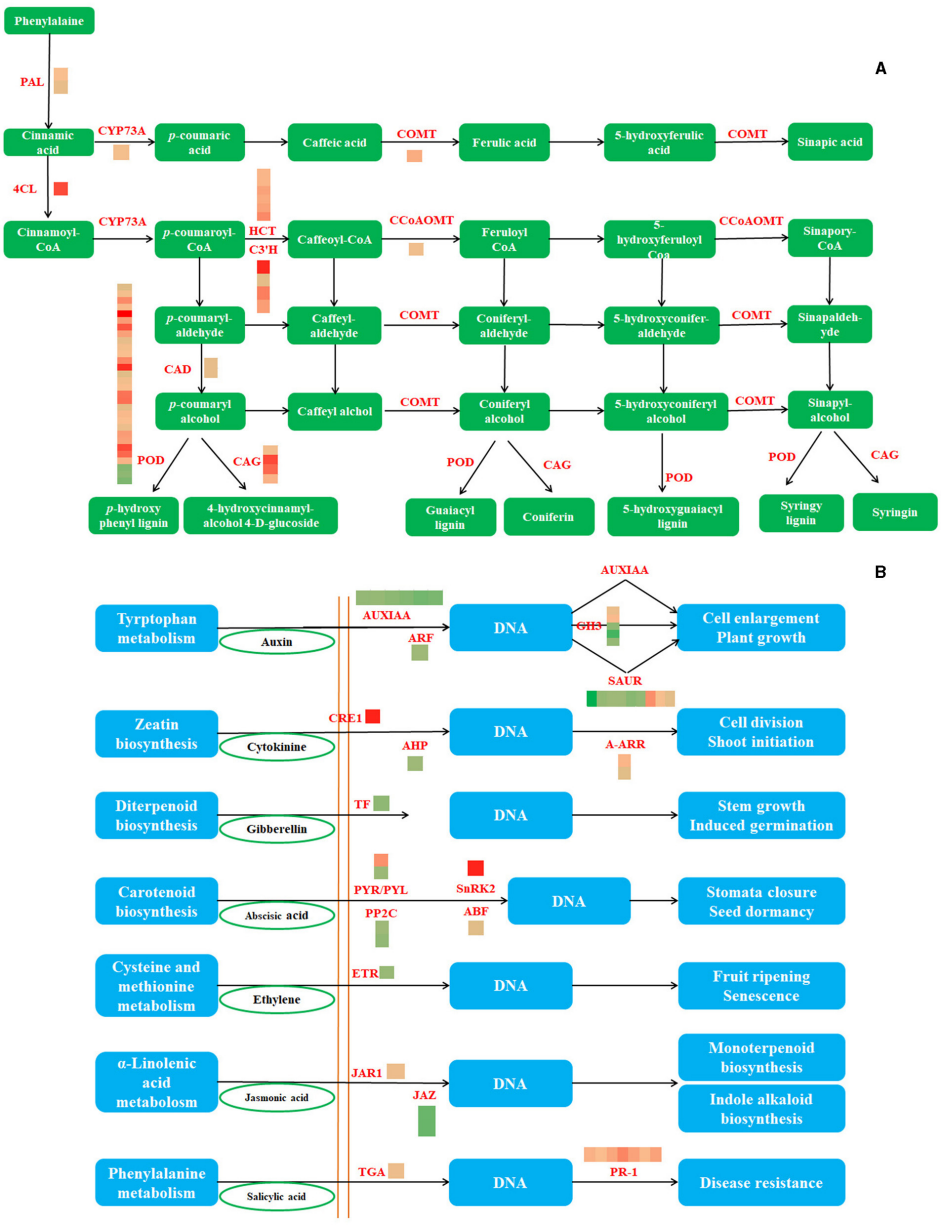
The phenylpropane biosynthesis pathway and the DEGs involved in this pathway **(A)** (PAL, phenylalanine ammonia-lyase; 4CL, 4-coumarate–CoA ligase; CYP73A, trans-cinnamate 4-monooxygenase; CAD, cinnamyl-alcohol dehydrogenase; COMT, caffeic acid 3-O-methyltransferase; POD, peroxidase; CCoAOMT, caffeoyl-CoA O-methyltransferase; CAG, coniferyl-alcohol glucosyltransferase) and The plant hormone signal transduction and the DEGs involved in this pathway **(B)** (AUXIAA, auxin-responsive protein IAA; ARF, auxin response factor;GH3, auxin-responsive GH3 gene family; SAUR, SAUR family protein; CRE1, cytokinin receptor; AHP, histidine-containing phosphotransfer protein; A-ARR, two-component response regulator ARR-A family; TF, phytochrome-interacting factor 4; PYR/PYL, abscisic acid receptor PYR/PYL family; PP2C, protein phosphatase 2C; SnRK2, serine/threonine-protein kinase SRK2; ABF, ABA-responsive element binding factor; ETR, ethylene receptor; JAR1, jasmonic acid-amino synthetase; JAZ, jasmonate ZIM domain-containing protein; TGA, transcription factor TGA; PR-1, pathogenesis-related protein 1).

Phenylpropanoid biosynthesis and plant hormone signal transduction were the most enriched pathways, with upregulated and downregulated DEGs, respectively. The three differential genes of phenylpropanoid biosynthesis were downregulated, whereas the others were upregulated. RT-qPCR analyses using eight genes associated with the phenylpropanoid pathway and 12 genes of plant hormone signal transduction were used to further confirm the RNA-seq results (Figure 4). As a result, consistent gene expression patterns were observed between qPCR and RNA-seq (Supplementary Figure 1).

### Metabolomic Data Analyses

**Figure 5:**
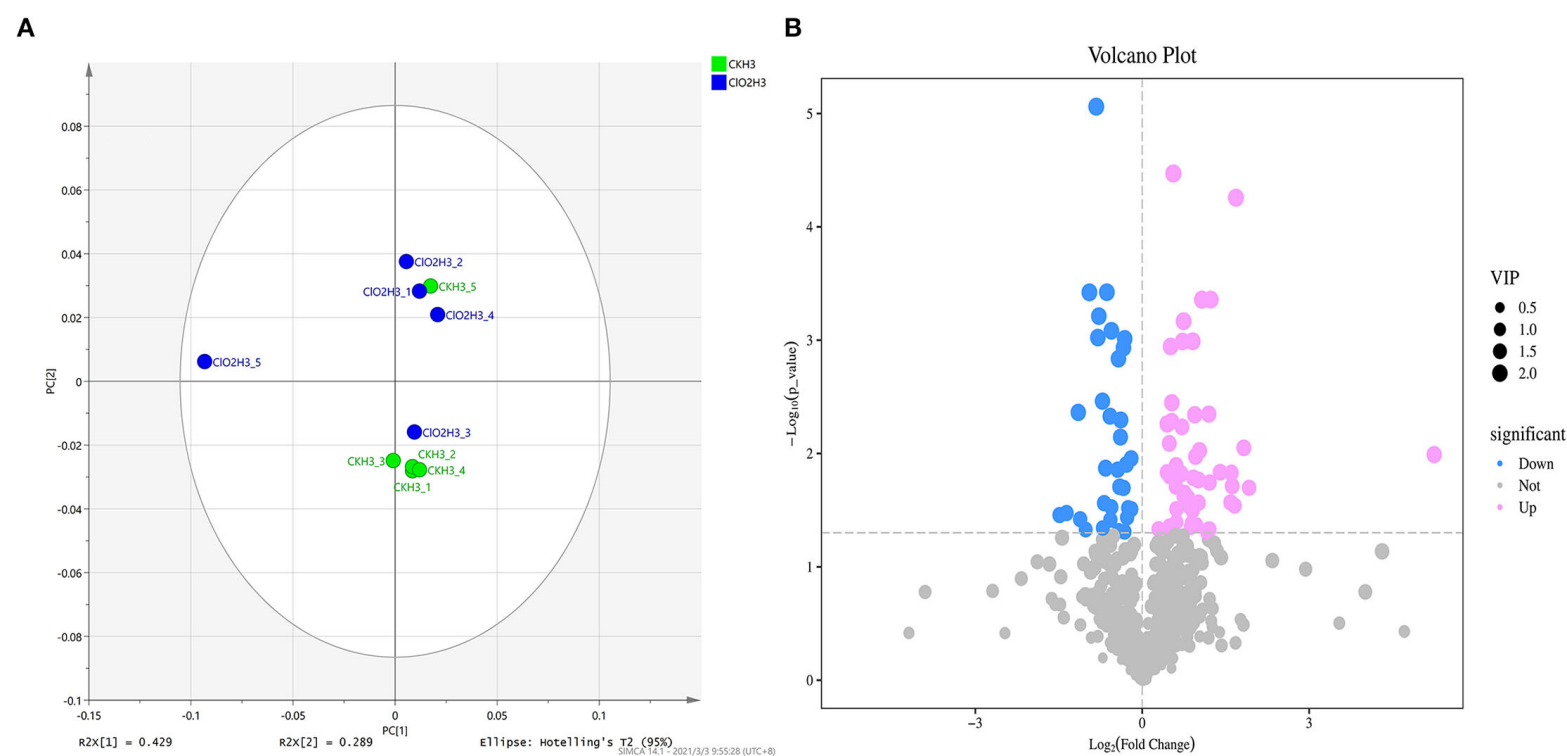
Differential metabolite screening PCA analysis **(A)** and volcanic map **(B)**.

The metabolomic assay identified a total of 932 metabolites. Principal components analysis evidently separated metabolites into treated and control groups, and samples with 95% confidence intervals (Hotelling's t-squared ellipse) were selected for further analyses to screen DAMs (Figure 5A). As a result, 85 DAMs were detected, with 33 and 52 DAMs being down- and upregulated, respectively, after ClO_2_ treatment (Figure 5B).

### Comparison of Metabolites

**Figure 6:**
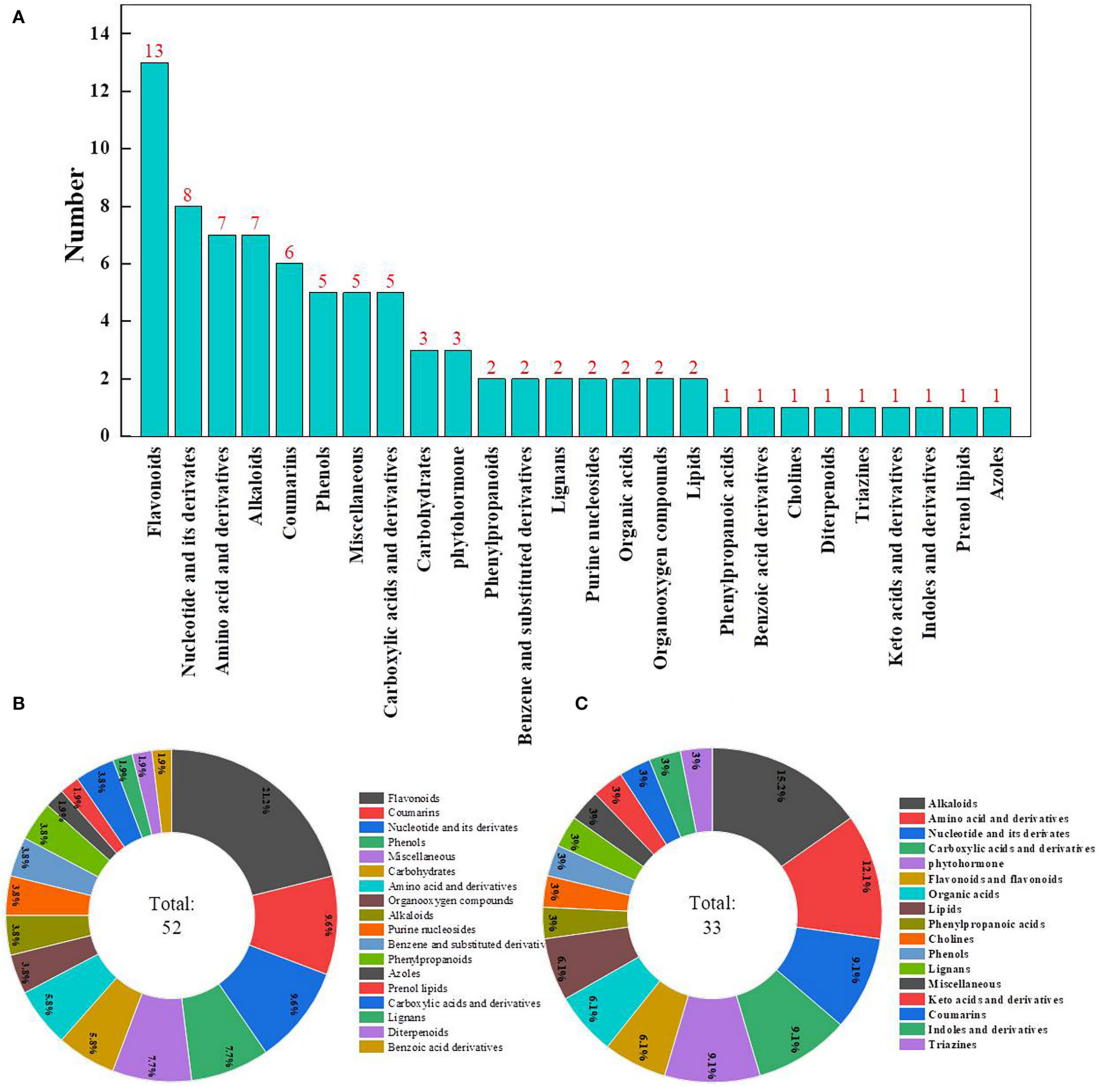
Differential metabolite **(A)**, upregulated **(B)**, and downregulated category **(C)**.

The most abundant DAMs identified were flavonoids, followed by nucleotides and their derivatives, amino acids and their derivatives, alkaloids, coumarins, phenols, miscellaneous, and carboxylic acids and their derivatives (Figure 6A). A total of 52 DAMs were upregulated, whereas 33 DAMs were downregulated in the pericarp of ClO_2_-treated tubers relative to that of control tubers. The most decreased metabolites were alkaloids, followed by amino acids and their derivatives, nucleotides and their derivatives, carboxylic acids and their derivatives, and phytohormones (Figure 6B). Most increased metabolites included flavonoids, followed by coumarins, nucleotides and their derivatives, phenols, and miscellaneous compounds (Figure 6C).

#### Integrated Transcriptomic and Metabolomic Analyses

**Figure 7:**
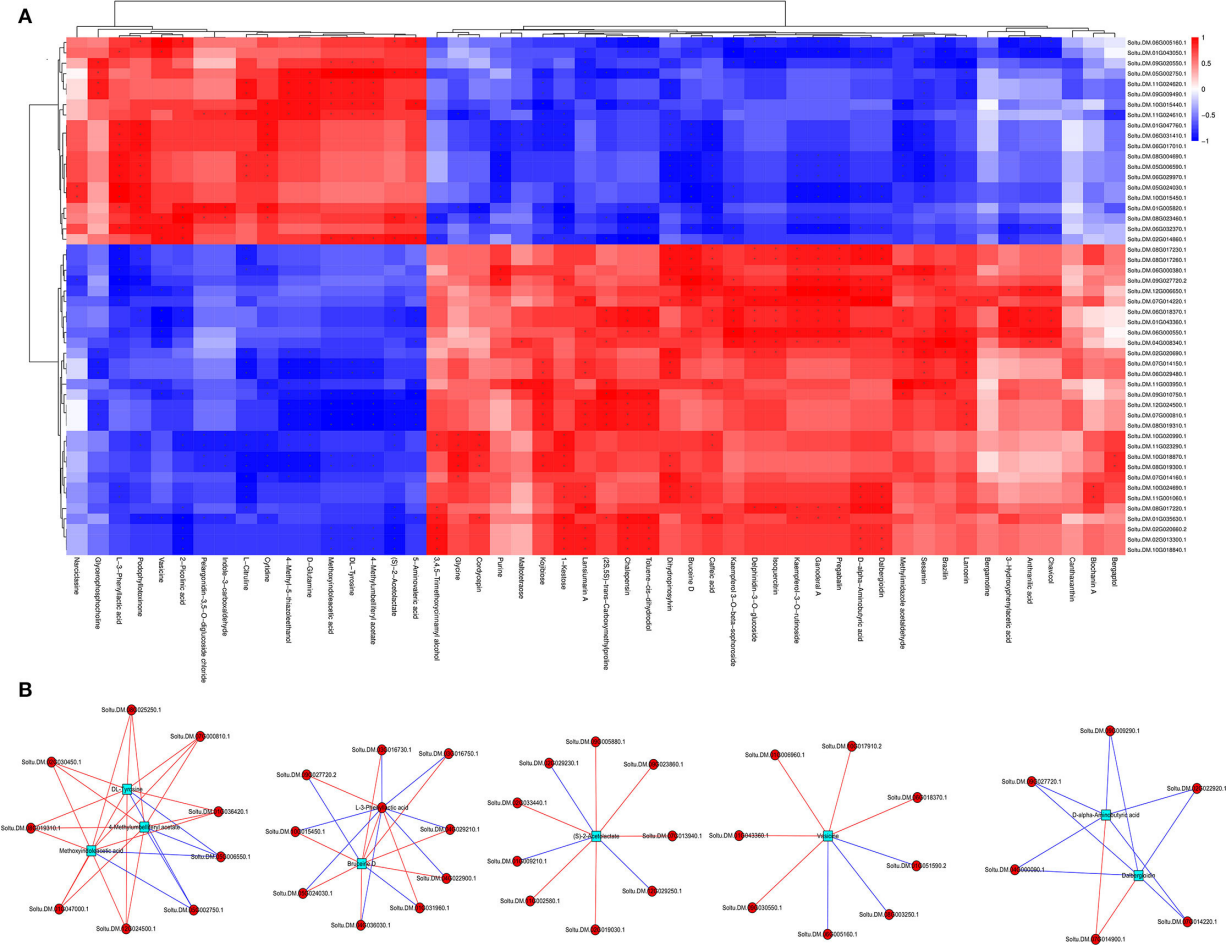
Correlation heat map **(A)** and network regulation map **(B)** between different genes and different metabolites.

The regulatory networks were analyzed by correlating DEGs and DAMs from each treatment. Results showed that multiple genes were positively or negatively regulated by multiple metabolites. For example, 17 DAMs were negatively or positively regulated by 20 or 30 DEGs, respectively, whereas 33 DAMs were negatively or positively regulated by 30 or 20 DEGs, respectively (Figure 7A). About three metabolites, 4-methylumbelliferyl acetate, methoxyindoleacetic acid, and DL-tyrosine, were strongly correlated with nine DEGs, with seven and two genes showing positive and negative correlations, respectively. Similarly, bruceine D and L-3-phenyllactic acid were significantly correlated with 10 DEGs, with eight and two genes showing positive and negative correlations, respectively, and 10 DEGs (three positively correlated, seven negatively correlated), respectively. In addition, (S)-2-acetolactate and vasicine were correlated with nine DEGs, with six and three genes showing positive and negative correlations, respectively, and eight DEGs (five positively correlated, three negatively correlated), respectively. Dalbergioidin and D-alpha-aminobutyric acid were markedly correlated with six DEGs, with one and five genes showing positive and negative correlations, respectively (Figure 7B). Notably, additional control networks were identified (Supplementary Figure 2).

## Discussion

Potato tuber sprouting caused commercial losses and results in increased content of solanine in the tuber, which poses a potential threat to human health (Liu et al., 2020). Transcriptomics and metabolomics can be used to better understand the mechanism of ClO_2_ -mediated repression of potato tuber sprouting. RNA-seq results identified 3,119 DEGs after ClO_2_ treatment, with the majority showing enrichment in phenylpropanoid biosynthesis, the MAPK signaling pathway plant, and plant hormone signal transmission pathways. Phenylpropanoid biosynthesis and MAPK signaling pathways were significantly activated, whereas the plant hormone signal transduction pathway was significantly inhibited. Consistently, ClO_2_ treatment could activate phenylpropane metabolism to improve apple tolerance (Zhao et al., 2011) and promote the accumulation of MAPK, which was shown to regulate browning in longan (Chumyam and Saengnil, 2020). Interestingly, ClO_2_ treatment hindered signal transduction during potato tuber sprouting. Phytohormones are crucial endogenous regulators of tuber sprouting, and dynamic changes in endogenous hormones related to stages of sprouting were observed in hormone-treated tubers (Aksenova et al., 2013). This suggests that ClO_2_ repressed potato sprouting by limiting plant hormone signal transduction. In addition, ClO_2_ could improve the stress tolerance of tubers by activating phenylpropanoid biosynthesis and MAPK signaling pathways. This could be because of the ability of ClO_2_ to induce the production of reactive oxygen species, which damage cell membranes, eventually leading to the obstruction of signal transduction during tuber sprouting.

Metabolites are important determinants of potato tuber sprouting (Aksenova et al., 2013). During the sprouting process, starch and protein degradation are initiated, and soluble sugars and amino acids are formed (Sonnewald, 2001). Metabolites are the final or intermediate regulatory products of the cell's biological processes (Pichersky and Lewinsohn, 2011), and their accumulation is controlled by numerous endogenous and exogenous factors. Plant growth and development are regulated by metabolites (Luca et al., 2012). Thus, integrated metabolomic and transcriptomic analyses can be used to identify key metabolic pathways and functional genes associated with plant growth and development (Matus, 2016; Wang et al., 2017). Potato sprouting is often accompanied by changes in a large number of metabolites (Sonnewald, 2001). Metabolomic results in this study revealed that most differentially upregulated metabolites associated with the repressing potato tuber sprouting were enriched in flavonoids and coumarins. In contrast, the most differentially downregulated metabolites were alkaloids, amino acids, and their derivatives. Flavonoids have health-promoting benefits because of their antioxidant capacity through cell signaling, pro-oxidative activity, or membrane characteristic mechanism (Mein et al., 2008). Potato tubers accumulate a high concentration of alkaloids, which might potentially compromise food safety because of their toxicity (Friedman, 2006). For example, the accumulation of glycoalkaloids can inhibit acetylcholinesterase activity and damage stomach cell membranes and has been associated with systemic side effects and gastrointestinal disturbance in people (Yamashoji and Matsuda, 2013). Alkaloid intake in small quantities can lead to gastrointestinal symptoms, such as abdominal pain, vomiting, and diarrhea, whereas higher alkaloid doses can cause acute poisoning or severe symptoms, such as coma, neurological disorders, cardiac failure, and paralysis (Mensinga et al., 2005). Our results showed that ClO_2_ could improve the antioxidant capacity and the safety of potato tubers by promoting the accumulation of flavonoids and inhibiting the synthesis of alkaloids, respectively (Deng et al., 2020; Wang et al., 2020).

Integrated metabolomic and RNA-seq were performed to determine the correlation between identified DEGs and DAMs. The results showed that different metabolites were regulated by different genes. For example, 4-methylumbelliferyl acetate and methoxyindoleacetic acid were regulated by nine genes, with seven and two genes as positive and negative regulators, respectively. The results suggest a complex regulatory mechanism between the changes in metabolite accumulation and gene expression abundance in potato tubers. Thus, we speculated that the mechanism of ClO_2_-repressed sprouting is achieved by a coordinated complex process involving several metabolic pathways, which inhibit signal transduction, plant hormones, and alkaloid synthesis in potato tubers, leading to repressed sprouting. The activity of ClO_2_ thus differs from other sprout inhibitors, such as chlorpropham, which is a mitotic inhibitor used to control tuber sprouting during storage (Vaughn and Lehnen, 1991). Essential oils instead act by damaging sprouts but do not really prevent their sprouting (Shukla et al., 2019). However, ClO_2_ is a safer and inexpensive sprout inhibitor compared to chlorpropham and essential oils, which are environmentally harmful and expensive (Fu et al., 2019). This study provides a basis for understanding the mechanism of ClO_2_-repressed sprouting; however, its specific regulatory roles still need further clarification.

## Conclusion

This study used ClO_2_-treated and control tubers to analyze the dynamic changes in metabolite accumulation and gene expression levels using UHPLC-MS and RNA-seq methods. The results revealed a total of 3,119 DEGs between the treated and control tubers. Most DEGs were enriched in phenylpropane metabolism, the MAPK signal pathway, and plant signal transduction pathways. ClO_2_ could inhibit potato tuber sprouting by altering plant signal transduction. Metabolomic analyses identified 85 differential metabolites, with flavonoids and coumarins as the most upregulated differential metabolites, and alkaloids and amino acids and their derivatives as the downregulated differential metabolites. The ClO_2_-mediated suppression of potato tuber sprouting is controlled by a complex regulatory network involving several different genes and metabolites.

## Data Availability Statement

The authors acknowledge that the data presented in this study must be deposited and made publicly available in an acceptable repository, prior to publication. Frontiers cannot accept an article that does not adhere to our open data policies.

## Author Contributions

XZ contributed to writing the original draft and writing, reviewing, and editing the manuscript. ML contributed to project administration and funding acquisition. ST contributed to supervision. SL contributed to formal analysis. JC contributed to project administration. XZ investigated the study. XW contributed to methodology. XG contributed to methodology software. JT contributed to data curation. YM contributed to resources. JS conceptualized the study. All authors contributed to the article and approved the submitted version.

## Conflict of Interest

The authors declare that the research was conducted in the absence of any commercial or financial relationships that could be construed as a potential conflictof interest.

## Publisher's Note

All claims expressed in this article are solely those of the authors and do not necessarily represent those of their affiliated organizations, or those of the publisher, the editors and the reviewers. Any product that may be evaluated in this article, or claim that may be made by its manufacturer, is not guaranteed or endorsed by the publisher.

## Supplementary Material

Supplementary Figure 1 Verification of some gene expression related to phenylpropane biosynthesis **(A)** and plant hormone signal transduction **(B)**. Bars indicate standard error (±SE). Asterisks indicate a significant difference (*p* < 0.05).

Supplementary Figure 2 The network regulation map between different genes and different metabolites.

Supplementary Table 1 Gene primer sequence of genes related to the synthesis of suberin and lignin.

Supplementary Table 2 Statistical sequencing data.

Supplementary Table 3 Statistical comparison efficiency of each sample.

## References

[B1] Aday M. S. Caner C. (2011). The applications of 'active packaging and chlorine dioxide' for extended shelf life of fresh strawberries. Pack. Technol. Sci. 24, 123–136. 10.1002/pts.918

[B2] Aksenova N. P. Sergeeva L. I. Konstantinova T. N. Golyanovskaya S. A. Kolachevskaya O. O. Romanov G. A. (2013). Regulation of potato tuber dormancy and sprouting. Russ. J. Plant Physiol. 60, 301–312. 10.1134/S1021443713030023

[B3] Atinut J. Jamnong U. Pathrapol L. Kobkiat S. (2019). Induced expression of NOX and SOD by gaseous sulfur dioxide and chlorine dioxide enhances antioxidant capacity and maintains fruit quality of 'daw' longan fruit during storage through H2O2 signaling-sciencedirect. Postharvest Biol. Technol. 156, 110938–110938. 10.1016/j.postharvbio.2019.110938

[B4] Campbell M. Gleichsner A. Alsbury R. Horvath D. Suttle J. (2010). The sprout inhibitors chlorpropham and 1, 4-dimethylnaphthalene elicit different transcriptional profiles and do not suppress growth through a prolongation of the dormant state. Plant Mol. Biol. 73, 181–189. 10.1007/s11103-010-9607-6 20135197

[B5] Chen Z. Zhu C. Han Z. (2011). Effects of aqueous chlorine dioxide treatment on nutritional components and shelf-life of mulberry fruit (*Morus alba* L.). J. Biosci. Bioeng. 111, 675–681. 10.1016/j.jbiosc.2011.01.010 21306948

[B6] Chumyam A. Saengnil K. (2020). Transient H2O2 induction by ClO2 fumigation alters prx-trx system and causes MAPK accumulation attenuating browning in harvested longan fruit. Chiang Mai Univ. J. Nat. Sci. 20:e2021013. 10.12982/CMUJNS.2021.013

[B7] Conte E. Imbroglini G. Bertolini P. Camoni I. (1995). Presence of sprout inhibitor residues in potatoes in relation to application techniques. J. Agricult. Food Chem. 43:2985–2987. 10.1021/jf00059a039

[B8] Corsini D. Stallknecht G. Sparks W. (1978). A simplified method for determining sprout inhibiting levels of chlorpropham (CIPC) in potatoes. J. Agricult. Food Chem. 26, 990–991. 10.1021/jf60218a030 670580

[B9] Deng Y. M. He M. Y. Feng F. Feng X. S. Zhang Y. Zhang F. (2020). The distribution and changes of glycoalkaloids in potato tubers under different storage time based on maldi-tof mass spectrometry imaging. Talanta, 221, 121453. 10.1016/j.talanta.2020.121453 33076076

[B10] Du J. Han Y. Linton R. H. (2003). Efficacy of chlorine dioxide gas in reducing *Escherichia coli* O157:H7 on apple surfaces, Food *Microbiology*. 20, 583–591. 10.1016/S0740-0020(02)00129-6

[B11] FDA (Food and Drug Administration). (1998). Bacteriological Analytical Manual, 8th Edition (Revision A) (Chapter 4) (Gaithersburg, MD: AOAC International), 4.20–4.26.

[B12] Foucart C. Paux E. Ladouce N. Grima-Pettenati J. Sivadon P. (2006). Transcript profiling of a xylem vs phloem cDNA subtractive library identifies new genes expressed during xylogenesis in Eucalyptus. New Phytologist, 170, 739–752. 10.1111/j.1469-8137.2006.01705.x 16684235

[B13] Friedman. (2006). Potato glycoalkaloids and metabolites: roles in the plant and in the diet. J. Agricult. Food Chem. 54, 8655–8681. 10.1021/jf061471t 17090106

[B14] Fu M.R. Zhang X.M. Jin T. Li B.Q. Zhang Z.Q. Tian S.P. (2019). Inhibitory of grey mold on green pepper and winter jujube by chlorine dioxide (ClO2) fumigation and its mechanisms. LWT. 100, 335–340. 10.1016/j.lwt.2018.10.092

[B15] Haas B. J. Papanicolaou A. Y assour M. Grabher M. Blood P. D. Bouden J. (2013). De novotranscript sequence reconstruction from RNA-seq using the Trinity platform for reference generation and analysis. *Nat*. Protoc. 8, 1494–1512. 10.1038/nprot.2013.084 23845962 PMC3875132

[B16] Huang Z. Tian S. L. Ge X. Zhang J. Li S. Q. Li M.. (2014). Complexation of chlorpropham with hydroxypropyl-β-cyclodextrin and its application in potato sprout inhibition. Carbohydrate Polymers, 107, 241–246. 10.1016/j.carbpol.2014.02.072 24702941

[B17] Kleinkopf G. Oberg N. Olsen N. (2003). Sprout inhibition in storage: Current status, new chemistries and natural compounds. Am. J. Potato Res. 80, 317–327. 10.1007/BF02854316

[B18] Kuhl C. Tautenhahn R. B. Ttcher C. Larson T. R. Neumann S. (2012). Camera: an integrated strategy for compound spectra extraction and annotation of liquid chromatography/mass spectrometry data sets. Anal.Chem. 84, 283–289. 10.1021/ac202450g 22111785 PMC3658281

[B19] Liu J, M. Wang S, S. Zheng Xu. Jin N. Lu J. Huang Y, T.. (2020). Antimicrobial activity against phytopathogens and inhibitory activity on Solanine in potatoes of the Endophytic Bacteria isolated from potato tubers. Front. Microbiol. 17, 570926. 10.3389/fmicb.2020.570926 33281766 PMC7705204

[B20] Livak K. J. Schmittgen T. D. (2001). Analysis of relative gene expression data using realtime quantitative PCR and the 2–ΔΔCT method. Methods. 25, 402–408. 10.1006/meth.2001.1262 11846609

[B21] Luca V. D. Satim V. Atsumi S. M. Fang Y. (2012). Mining the biodiversity of plants: a revolution in the making. Science. 336, 1658. 10.1126/science.1217410 22745417

[B22] Matus J. T. (2016). Transcriptomic and metabolomic networks in the grape berry illustrate that it takes more than flavonoids tofight against ultraviolet radiation. Front. Plant Sci. 7, 1337. 10.3389/fpls.2016.01337 27625679 PMC5003916

[B23] Mein J.R. Lian F. Wang X.D. (2008). Biological activity of lycopene metabolites: implications for cancer prevention. Nutr. Rev. 66, 667–683. 10.1111/j.1753-4887.2008.00120.x 19019036 PMC6824483

[B24] Mensinga T. T. Sips A. Rompelberg C. Twillert K. V. Meulenbelt J. Top H.. (2005). Potato glycoalkaloids and adverse effects in humans: an ascending dose study. Regul. Toxicol. Pharmacol. 41, 66–72. 10.1016/j.yrtph.2004.09.004 15649828

[B25] Nicot N. Hausman J. F Hoffmann L. Danièle Evers. (2005). Reference gene selection for rt-qpcrnormalization in potato during biotic and abiotic stress. J. Exp. Bot. 56, 2907–2914. 10.1093/jxb/eri285 16188960

[B26] Niu R. X. Hui C. X. Yin-Hua T. U. Jin H. (2009). Effects of chlorine dioxide (ClO_2_) treatment on fresh-keeping and storage quality of qinmei kiwifruit. Sci. Technol. Food Indus. 1, 289–292. 10.13386/j.issn1002-0306.2009.01.072

[B27] Pao S. Kelsey D. F. Long W. (2009). Spray washing of tomatoes with chlorine dioxide to minimize salmonella on inoculated fruit surfaces and cross-contamination from revolving brushes. J. Food Protect. 72, 2448-2452. 10.4315/0362-028X-72.12.2448 20003724

[B28] Pichersky E. Lewinsohn E. (2011). Convergent evolution in plant specialized metabolism. Ann. Rev. Plant Biol. 62, 549–566. 10.1146/annurev-arplant-042110-103814 21275647

[B29] Sawada Y. J. Kenji A. Akane S. Ayuko K. Hitomi O. Tetsuya S.. (2009). Widely targeted metabolomics based on large-scale MS/MS data for elucidating metabolite accumulation patterns in plants. Plant Cell Physiol. 50, 37–47. 10.1093/pcp/pcn183 19054808 PMC2638709

[B30] Shukla S. Pandey S. S. Chandra M. Pandey A. Bharti N. Barnawal D. (2019). Application of essential oils as a natural and alternate method for inhibiting and inducing the sprouting of potato tubers. Food Chem. 284, 171–179. 10.1016/j.foodchem.2019.01.079 30744843

[B31] Sonnewald U. (2001). Control of potato tuber sprouting. Trends Plant Sci. 6, 333–335. 10.1016/S1360-1385(01)02020-9 11495763

[B32] Teper-Bamnolker P. Dudai N. Fischer R. Belausov E. Zemach H. Shoseyov O.. (2010). Mint essential oil can induce or inhibit potato sprouting by differential alteration of apical meristem. Planta. 232, 179–186. 10.1007/s00425-010-1154-5 20390295

[B33] Tweddell R. J. Boulanger R. Arul J. (2003). Effect of chlorine atmospheres on sprouting and development of dry rot, soft rot and silver scurf on potato tubers. Postharvest Biol. Technol. 28, 445–454. 10.1016/S0925-5214(02)00205-3

[B34] Vaughn K. C. Lehnen L. P. (1991). Mitotic disrupter herbicides. Weed Sci. 39, 450–457. 10.1017/S0043174500073215

[B35] Wang Z. K. Ma R. Zhao M. S. Wang F. F. Zhang N. Si H. J. (2020). NO and ABA interaction regulates tuber dormancy and sprouting in potato. Front. Plant Sci. 11, 311. 10.3389/fpls.2020.00311 32322258 PMC7156616

[B36] Wang Z. R. Cui Y. Y. V ainstein A. Chen S. W. Ma H. Q. (2017). Regulation of fig (*Ficus carica* L.) fruit color: metabolomic and transcriptomic analyses of the flavonoid biosynthetic pathway. Front. Plant Sci. 8, 1990. 10.3389/fpls.2017.01990 29209349 PMC5701927

[B37] Woolfson K. N. Haggitt M. L. Zhang Y. Kachura A. Bjelica A. Rincon M. A. R.. (2018). Differential induction of polar and non-polar metabolism during wound-induced suberization in potato (*Solanum tuberosum* L.) tubers. Plant J. Cell Mol. Biol. 93, 931-942. 10.1111/tpj.13820 29315972

[B38] Wu B. Li X. Hu H. Liu A. Chen W. (2011). Effect of chlorine dioxide on the control of postharvest diseases and quality of litchi fruit. Afr. J. Biotechnol. 10, 6030-6039.

[B39] Yamashoji S. Matsuda T. (2013). Synergistic cytotoxicity induced by α-solanine and α-chaconine. Food Chem. 141, 669-674. 10.1016/j.foodchem.2013.03.104 23790833

[B40] Zhang Z. M. Tong X. Peng Y. Ma P. Zhang M. J. Lu H. M.. (2015). Multiscale peak detection in wavelet space. Analyst. 140, 7955-7964. 10.1039/C5AN01816A 26514234

[B41] Zhao M. H. Rao J. P. Xing F. C. Xia Y. Y. (2011). Fresh-keeping effect of pre-harvest chlorine dioxide treatment of ‘Red Fuji' apple. Food Sci. 32, 352–356.

