# Corrigendum: Integrated analysis of transcriptome and metabolome reveals the mechanism of chlorine dioxide repressed potato (*Solanum tuberosum* L.) tuber sprouting

**DOI:** 10.3389/fpls.2024.1373758

**Published:** 2024-03-15

**Authors:** Xiaoyuan Zheng, Mei Li, Shilong Tian, Shouqiang Li, Jianxin Chen, Xuejiao Zhang, Xiaohua Wu, Xia Ge, Jiachun Tian, Yuwen Mu, Juan Song

**Affiliations:** ^1^ Agricultural Product Storage and Processing Research Institute, Gansu Academy of Agricultural Sciences, Lanzhou, China; ^2^ College of Food Science and Engineering, Gansu Agricultural University, Lanzhou, China; ^3^ Gansu Innovation Center of Fruit and Vegetable Storage and Processing, Lanzhou, China

**Keywords:** potato tuber, chlorine dioxide, repression of sprout, transcriptome, metabolome

In the published article, there was an error in **Figure 4** as published. In **Figure 4B**, ‘Zeatin biosynthesis’ and ‘Stem growth induced germination’ appeared twice. The corrected **Figure 4** and its caption appear below.

**Figure 4:**
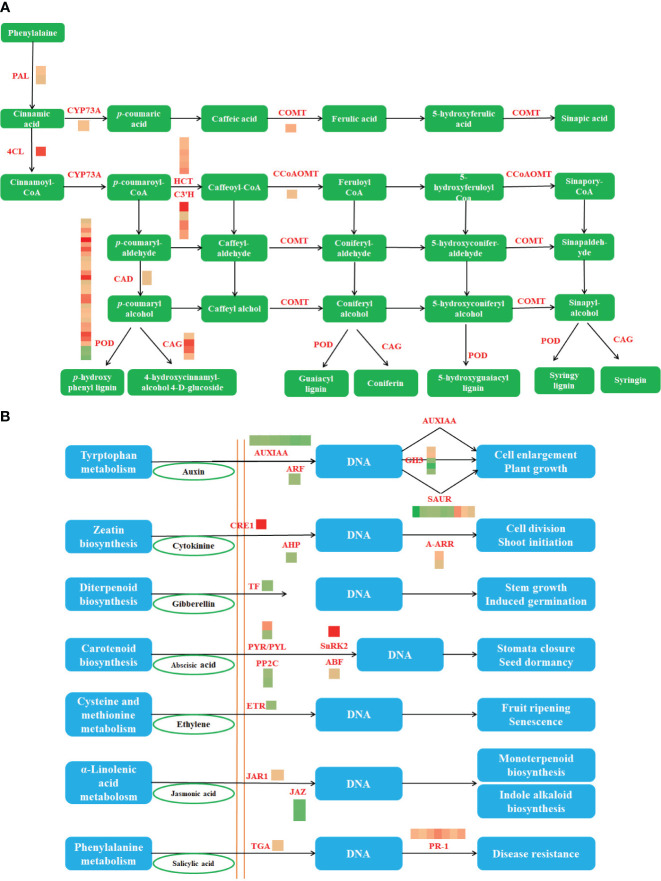
The phenylpropane biosynthesis pathway and the DEGs involved in this pathway **(A)** (PAL, phenylalanine ammonia-lyase; 4CL, 4-coumarate–CoA ligase; CYP73A, trans-cinnamate 4-monooxygenase; CAD, cinnamyl-alcohol dehydrogenase; COMT, caffeic acid 3-O-methyltransferase; POD, peroxidase; CCoAOMT, caffeoyl-CoA O-methyltransferase; CAG, coniferyl-alcohol glucosyltransferase) and The plant hormone signal transduction and the DEGs involved in this pathway. **(B)** (AUXIAA, auxin-responsive protein IAA; ARF, auxin response factor; GH3, auxin-responsive GH3 gene family; SAUR, SAUR family protein; CRE1, cytokinin receptor; AHP, histidine-containing phosphotransfer protein; A-ARR, two-component response regulator ARR-A family; TF, phytochrome-interacting factor 4; PYR/PYL, abscisic acid receptor PYR/PYL family; PP2C, protein phosphatase 2C; SnRK2, serine/threonine-protein kinase SRK2; ABF, ABA-responsive element binding factor; ETR, ethylene receptor; JAR1, jasmonic acid-amino synthetase; JAZ, jasmonate ZIM domain-containing protein; TGA, transcription factor TGA; PR-1, pathogenesis-related protein 1).

The authors apologize for this error and state that this does not change the scientific conclusions of the article in any way. The original article has been updated.

