# Supplementary material for: Integrated Analysis of Transcriptome and Metabolome Reveals the Mechanism of Chlorine Dioxide Repressed Potato (Solanum tuberosum L.) Tuber Sprouting
Source: Front Plant Sci. 2022 May 23;13:887179. doi: 10.3389/fpls.2022.887179 (PMC9175755; doi:10.3389/fpls.2022.887179)
Supplement: Supplementary Figure 1 — Verification of some gene expression related to phenylpropane biosynthesis (A) and plant hormone signal transduction (B). Bars indicate standard error (±SE). Asterisks indicate a significant difference (p < 0.05). [file Data_Sheet_1.docx]

**Supplementary Materials**

**Supplementary Fig. 1.** Verification of some gene expression related to phenylpropane biosynthesis (A) and plant hormone signal transduction (B). Bars indicate standard error (±SE). Asterisks indicate significant difference (*P*< 0.05).

**Supplementary Fig. 2.** The network regulation map (B) between different genes and different metabolites.

**Supplementary Table. 1.** Statistical of sequencing data.

**Supplementary Table. 2.**Statistical of comparison efficiency of each sample.

**Supplementary Table. 3.** Gene primer sequence of genes related to the synthesis of suberin and lignin.

Supplementary Fig. 1.


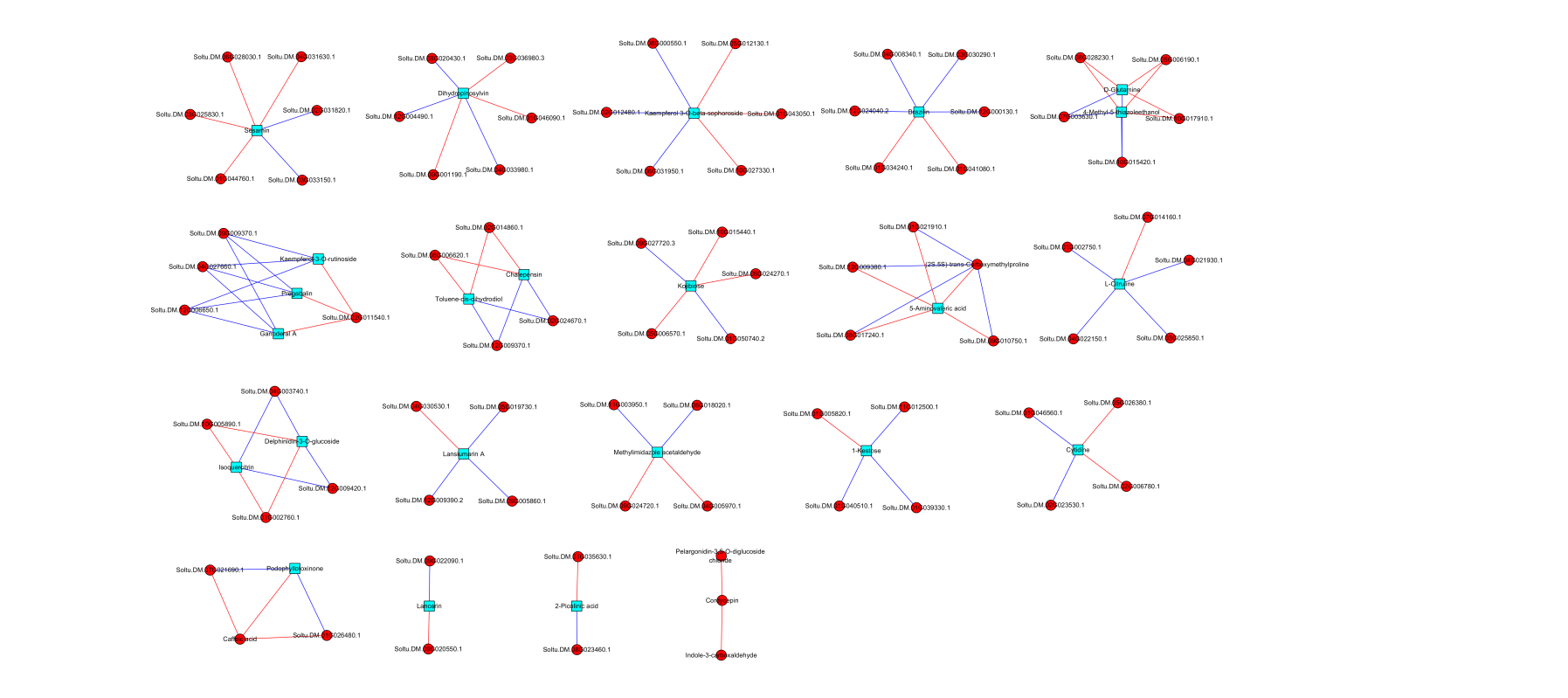


Supplementary Fig. 2.

| Sample | ReadSum | BaseSum | GC (%) | N (%) | Q20 (%) | CycleQ20 (%) | Q30 (%) |
| --- | --- | --- | --- | --- | --- | --- | --- |
| CK-1 | 19954863 | 5986458900 | 42.92 | 0 | 97.85 | 100 | 93.75 |
| CK-2 | 21232943 | 6369882900 | 43.12 | 0 | 97.79 | 100 | 93.72 |
| CK-3 | 20859956 | 6257986800 | 43.15 | 0 | 97.84 | 100 | 93.78 |
| ClO2-1 | 23104861 | 6931458300 | 42.95 | 0 | 97.64 | 100 | 93.33 |
| ClO2-2 | 24982040 | 7494612000 | 42.86 | 0 | 97.59 | 100 | 93.23 |
| ClO2-3 | 25784845 | 7735453500 | 42.69 | 0 | 97.54 | 100 | 93.21 |

Supplementary Table. 1.

| CK-1 | CK-2 | CK-3 | ClO_2_-1 | ClO_2_-2 | ClO_2_-3 |
| --- | --- | --- | --- | --- | --- |
| not aligned | 4569951  (10.49%) | 4598523  (10.41%) | 4355365  (10.75%) | 4431347  (10.13%) | 4967442  (9.90%) |
| reads aligned | 38978691  (89.51%) | 39586917  (89.59%) | 36151465  (89.25%) | 39301759  (89.87%) | 45190468  (90.10%) |

Supplementary Table. 2.

| Genes | Gene ID. | Primer sequence (5’–3’) | Product length (bp) |
| --- | --- | --- | --- |
| *StPAL1* | Soltu.DM.05G026870.1 | Forward: GCGTCTGGTGACTTGGTAC  Reverse: GCTAATCCTTCTTTGGGCT | 167 |
| *StPAL2* | Soltu.DM.10G020990.1 | Forward: ATGGCTTCATACTGTTCGG  Reverse: ACTGCTTCAGCTGTTTTTC | 131 |
| *St4CL* | Soltu.DM.07G003850.1 | Forward: TATTGATGCTGCTGTTGTC  Reverse: TCGATTTATTCGCTTGTAA | 151 |
| *StCAD* | Soltu.DM.12G008570.2 | Forward: GTTACTAAAGGGGGATACT  Reverse: TCCAACGACACCTAAAGAT | 177 |
| *StPOD1* | Soltu.DM.01G001840.1 | Forward: TGCCCTGGTGTTGTTTCTT  Reverse: GCTCCACTTCTGTTTGCTG | 131 |
| *StPOD2* | Soltu.DM.01G001850.1 | Forward: CGGTGTTGTATCTTGTGCT  Reverse: TTGTTGGTGAATTGTGGTC | 189 |
| *StPOD3* | Soltu.DM.02G034250.1 | Forward: TCTCGGGTTGCTGGAAAAT  Reverse: GGGTTGGATGGGGTAAATG | 179 |
| *StPOD4* | Soltu.DM.09G006170.2 | Forward: ATGAAAACAGGAAGGAAAG  Reverse: GAGTGAGCCCCTAGTAGAG | 155 |
| *StAUXIAA1* | Soltu.DM.09G020550.1 | Forward: AGATTAGGTTTGCCTGGGA  Reverse: TTTGGTGGAGGAGGTGAGT | 125 |
| *StAUXIAA2* | Soltu.DM.09G020550.2 | Forward: ATTAGGTTTGCCTGGGATA  Reverse: GTGGAGGAGGTGAGTCTTG | 119 |
| *StARF* | Soltu.DM.02G016930.2 | Forward: CTTCAAAGTTCCAGGCTAT  Reverse: TTCCTTCCTACTGATGTCC | 137 |
| *StCRE1* | Soltu.DM.04G003490.2 | Forward: AAGATTGATAAATGAAGTG  Reverse: TCAGAGAATAAAGAGAGAA | 127 |
| *StAHP* | Soltu.DM.03G035760.1 | Forward: GACTCTGCTCGGTTGATCC  Reverse: TGCCCTTCTACATTTCCTG | 191 |
| *StTF* | Soltu.DM.07G014300.3 | Forward: AACCAAACACATAAAAAGC  Reverse: AGATAAGAAAGGGGAACAA | 189 |
| *StPP2C1* | Soltu.DM.07G012130.1 | Forward: AGTCAAACACTTACAGCCC  Reverse: TTCCATTATGTGAATCCTC | 139 |
| *StPP2C2* | Soltu.DM.07G012130.7 | Forward: ATAAACAGGGAAAATTGAT  Reverse: GTTAGGTTCTGATAGCAAG | 107 |
| *StETR* | Soltu.DM.09G026120.1 | Forward: ATGTTATGCTATCCTCGTT  Reverse: TGCTTGATTCTGTTCTGTC | 175 |
| *StJAZ1* | Soltu.DM.03G036980.3 | Forward: AAGCAAATCCAGTCACAGG  Reverse: AAAGTAGACCCCAAACGTC | 137 |
| *StJAZ2* | Soltu.DM.07G012950.3 | Forward: TAAAGAAGAACACAAATCC  Reverse: AATCATCAAAAACAATCAC | 161 |
| *StTGA* | Soltu.DM.04G028540.4 | Forward: GAAGAGCAACAAAAAAAGA  Reverse: AAGTAAAACACGTCAGCCT | 155 |
| *Efla** |  | Forward: CAAGGATGACCCAGCCAAG  Reverse: TTCCTTACCTGAACGCCTGT | 133 |

* From Nicot and Evers (2005).

**Supplementary Table. 3.**
